# Derivation and validation of a model predicting the likelihood of vaginal birth following labour induction

**DOI:** 10.1186/s12884-019-2232-8

**Published:** 2019-04-16

**Authors:** Sepand Alavifard, Kennedy Meier, Yonatan Shulman, George Tomlinson, Rohan D’Souza

**Affiliations:** 1 Division of Maternal-Fetal Medicine, Department of Obstetrics & Gynaecology, Mount Sinai Hospital, University of Toronto, Toronto, ON Canada; 2 Faculty of Medicine, University of Toronto, Toronto, ON Canada; 3 Department of Medicine, University Health Network and Mount Sinai Hospital, Toronto, ON Canada; 4 Institute of Health Policy, Management and Evaluation, University of Toronto, Toronto, ON Canada; 5 Lunenfeld Tanenbaum Research Institute, Mount Sinai Hospital, Toronto, ON Canada

**Keywords:** Pregnancy, Induction of labour, Prediction tool, Successful induction, Vaginal birth

## Abstract

**Background:**

There is high-quality evidence supporting induction of labour (IOL) for a number of maternal and fetal indications. However, one fifth of inductions fail to result in vaginal births, requiring cesarean deliveries. This has negative clinical, emotional and resource implications. The importance of predicting the success of labour induction to enable shared decision-making has been recognized, but existing models are limited in scope and generalizability. Our objective was to derive and internally validate a clinical prediction model that uses variables readily accessible through maternal demographic data, antenatal history, and cervical examination to predict the likelihood of vaginal birth following IOL.

**Methods:**

Data was extracted from electronic medical records of consecutive pregnant women who were induced between April and December 2016, at Mount Sinai Hospital, Toronto, Canada. A multivariable logistic regression model was developed using 16 readily accessible variables identified through literature review and expert opinion, as predictors of vaginal birth after IOL. The final model was internally validated using 10-fold cross-validation.

**Results:**

Of the 1123 cases of IOL, 290 (25.8%) resulted in a cesarean delivery. The multivariable logistic regression model found maternal age, parity, pre-pregnancy body mass index and weight, weight at delivery, and cervical dilation at time of induction as significant predictors of vaginal delivery following IOL. The prediction model was well calibrated (Hosmer-Lemeshow χ2 = 5.02, *p* = 0.76) and demonstrated good discriminatory ability (area under the receiver operating characteristic (AUROC) curve, 0.81 (95% CI 0.78 to 0.83)). Finally, the model showed good internal validity [AUROC 0.77 (95% CI 0.73 to 0.82)].

**Conclusions:**

We have derived and internally validated a well-performing clinical prediction model for IOL in a large and diverse population using variables readily accessible through maternal demographic data, antenatal history, and cervical examination. Once prospectively validated in diverse settings, and if shown to be acceptable to pregnant women and healthcare providers as well as clinically and cost-effective, this model has potential for widespread use in clinical practice and research for enhancing patient autonomy, improving induction outcomes, and optimizing allocation of resources.

**Electronic supplementary material:**

The online version of this article (10.1186/s12884-019-2232-8) contains supplementary material, which is available to authorized users.

## Key message

We have derived and internally validated a prediction model, having built on limitations of existing models, to accurately determine the success of vaginal birth following IOL, with the intent of improving outcomes, enhancing shared decision-making and enabling optimal resource allocation.

## Background

Induction of labour (IOL) refers to the artificial initiation of labour undertaken when the benefits of delivery are deemed to outweigh the risk of awaiting the spontaneous onset of labour [1, 2]. It is a common obstetric intervention that precedes approximately one in five births [1, 3], and numbers are expected to increase with emerging evidence recommending IOL for a variety of obstetric and medical indications [4–6]. Unfortunately, one-fifth of IOLs fail to result in vaginal birth, requiring unplanned cesarean deliveries, often after arduous labours [1]. Apart from having a profound impact on birth experiences of women, IOL has clinical and cost implications due to their unpredictable duration and likelihood of success, as well as higher maternal and neonatal complications with failed IOL resulting in unplanned cesarean deliveries [7]. Predicting the likelihood of vaginal birth following IOL has become important in this era of personalized medicine, not just from the point of view of women being aware of the chances of success of an intervention, but also to ensure optimal allocation of healthcare resources.

Although the Bishop score is commonly used to predict the success of IOL [1, 8], it fares poorly as a prediction tool [9]. Published prediction models are often limited in their scope and generalizability [10, 11], or use complex clinical, radiologic and biochemical variables that are difficult to adapt to diverse clinical settings [12, 13]. The objective of our study was to derive and internally validate a clinical prediction model that used variables readily available from maternal demographic data, antenatal history, and cervical examination to predict the likelihood of vaginal birth following IOL. Such a model could be developed into a tool that empowers shared decision-making, and optimizes healthcare resource utilization.

## Methods

### Study Design & Population

We conducted a retrospective review of electronic medical record data for consecutive pregnancies in 2016 at Mount Sinai Hospital, a tertiary-level center and Canada’s largest obstetric unit. Pregnant women with a single live intrauterine fetus in the cephalic presentation and no history of prior cesarean delivery, induced after 33 completed weeks of gestation were included, regardless of the indication for induction or cervical favorability. We excluded IOLs performed for intrauterine fetal demise or lethal/major congenital malformations, planned IOLs that presented in spontaneous labour, cases where the decision to induce labour was changed prior to the date of IOL for any reason and those where the time of initiating IOL was missing. We had originally intended to include women with prior cesarean deliveries. However, at our institution, as is the case with many institutions in North America, labour inductions following cesarean deliveries were extremely infrequent. Women with prior cesarean deliveries were therefore excluded from the study. This study was conducted in accordance with the Transparent Reporting of a Multivariable Prediction Model for Individual Prognosis or Diagnosis (TRIPOD) Statement (Additional file 1) [14].

### Outcome, predictor variables, and population characteristics

With no evidence-based or widely accepted definition of successful IOL [15], the outcome of interest was defined as vaginal delivery. Predictor variables had to be easily accessible through maternal demographic data, antenatal history, or cervical examination. In order to identify predictor variables, a systematic review of the literature was conducted and 16 predictor variables were identified [10]. A full list of all extracted variables and their definitions can be found in the supplementary material (Additional file 2). In addition to predictor variables, information on the method of induction, the initial method where multiple methods were used, and indication for induction were also extracted.

### Sample size

Of the 16 included variables, nine were continuous and seven were categorical. Within the seven categorical variables, there were a total of 20 levels, requiring 13 parameters. Therefore, there were a total of at least 29 parameters to be estimated in a model. To obtain stable estimates of parameters, a minimum suggested sample size is 10 events per variable [16]. This leads to a requirement of a minimum of 290 events (vaginal deliveries) or non-events (cesarean deliveries), whichever smaller, in the final dataset. With a cesarean delivery rate of approximately 25% after induction at Mount Sinai Hospital, we calculated a full sample size of 1200 cases of IOL. We decided a priori, that we would stop collecting data when we had the required 290 cesarean deliveries, regardless of the total numbers recruited. Data collection was commenced in a reverse chronological order from December 31, 2016 until the sample size was arrived at.

### Missing data

Once data collection was complete, the first step was to assess missingness. Where possible, other sources such as paper charts, ultrasound records, and triage visits were the first recourse to locate missing data. For variables where ≤10% of data were missing, we attempted to evaluate whether this missingness was at random or not, based on visual assessment of data. If at random, single imputation modeling with predictive mean matching was used to substitute missing value with estimates [17]. If not at random, missingness was managed on a case-by-case basis and explicitly described. The impact of imputation modeling on the developed prediction model was assessed in a sensitivity analysis in which the performance of the final model was assessed using data only from patients with complete information for variables included in the final model.

Variables with missing data for > 10% of women were omitted from model development [18]. We decided a priori to assess the potential predictive value of variables omitted for this reason by conducting a second sensitivity analysis. In this analysis, a prediction model was developed using data from patients with complete information for all 16 potential predictor variables (complete-case analysis).

### Statistical analyses

A multivariable logistic regression model was developed using identified predictor variables with ≤10% missingness. To account for possible non-linearity, continuous variables were added as third degree polynomials. Predictors were then excluded by backward-elimination based on the Akaike Information Criterion (AIC), a criterion approach based on the residual sum of squares, used to compare non-nested models to identify an optimal subset of predictors. Of the remaining variables, those with *p* > 0.20 were later removed from the model [19]. The discriminatory ability of the prediction model was assessed using the area under the receiver operating characteristic (AUROC) curve and the *pROC* library in R was used to compute 95% confidence intervals [20]. The calibration of the prediction model was assessed using a calibration plot [21] and the Hosmer-Lemeshow goodness-of-fit test (*p* < 0.05 was taken to indicate a statistically significant lack of fit). Internal validity of the prediction model was evaluated using 10-fold cross-validation. All analyses were done using R version 3.3.1 (R Foundation for Statistical Computing, Vienna, Austria).

### Ethical approval

This study was approved by the Mount Sinai Hospital Research Ethics Board (REB #17–0037-C). The approval included permission to access electronic medical records of patients that met the study’s predefined selection criteria.

## Results

### Study population

**Fig. 1:**
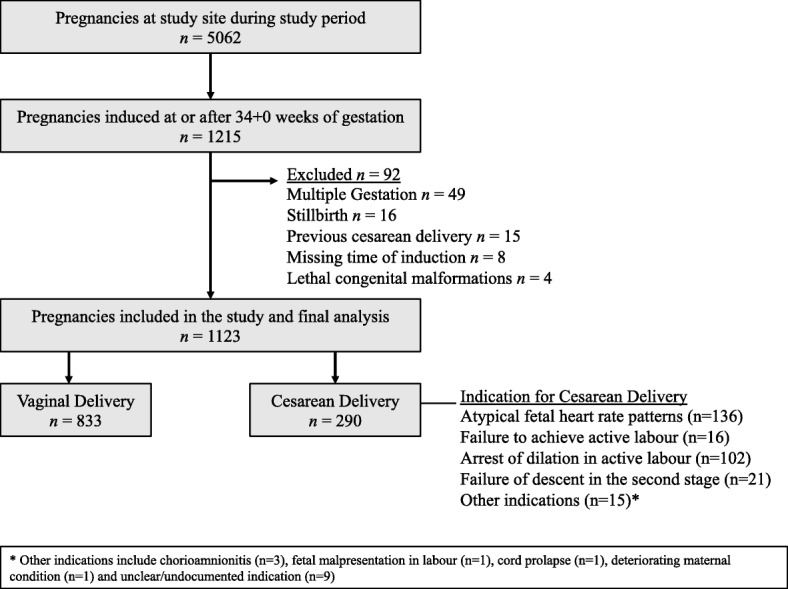
Patient Selection Flow Chart

A total of 1123 consecutive IOLs took place between April 5, 2016 and December 31, 2016 of which 833 (74.2%) resulted in vaginal delivery. Most cesarean deliveries were performed for concerns with fetal heart rate patterns in labour (136/290) and arrest of dilation in the active phase of the first stage of labour (102/290), and only a small minority for failure to achieve active labour (Fig. 1). Population characteristics and proportion of missing data for each variable are summarized in Table 1.

**Table 1:** Baseline Characteristics of Study Population

	Characteristic	Cohort (*N* = 1123)*	Missing (%)
Maternal Characteristics	Maternal Age (in years)	33.9 ± 4.8	0
	Height (in centimeters)	164.8 ± 7.2	3.1
	Weight (pre-pregnancy) (kilograms)	67.6 ± 17.8	4.9
	Body Mass Index (pre-pregnancy) (kg/m^2^)	24.9 ± 6.1	6.2
	Weight (at-delivery) (kilograms)	82.4 ± 18.6	4.3
	Body Mass Index (at-delivery) (kg/m^2^)	30.4 ± 6.3	5.8
	Weight Gain (kilograms)	14.7 ± 6.5	7.5
Obstetrical History	Nulliparous *n (%)*	703 (62.6)	0
	Primigravida *n (%)*	504 (44.9)	0
Cervical Examination	Dilation (centimeters) *median and interquartile range*	1.7 ± 1.32	8.4
	Consistency *n (%)*		49.2
	Firm	24 (4.2)	
	Medium	48 (8.4)	
	Soft	498 (87.4)	
	Position *n (%)*		47.2
	Anterior	148 (25)	
	Mid	88 (14.8)	
	Posterior	357 (60.2)	
	Effacement *n (%)*		22.7
	0–29%	181 (20.8)	
	30–49%	74 (8.5)	
	50–69%	340 (39.2)	
	> 70%	273 (31.5)	
	Fetal Station *n (%)*		46.7
	-3	233 (39.0)	
	-2	321 (53.7)	
	-1	36 (6.0)	
	0	8 (1.3)	
Labour Characteristics	Indication for Induction		0
	Fetal^†^ *n (%)*	272 (24.2)	
	Maternal Indication for Induction^‡^ *n (%)*	537 (47.8)	
	Social/Geographical/Other *n (%)*	314 (28.0)	
	Primary Method of Induction *n (%)*		0
	Artificial rupture of membranes	334 (29.7)	
	Cervidil	42 (3.7)	
	Foley Catheter	139 (12.4)	
	Oxytocin	215 (19.1)	
	Prostaglandin Gel/Tablet	393 (35.0)	
	Gestational Age	39.4 ± 1.4	0
	Mode of Delivery *n (%)*		0
	Cesarean Delivery	290 (25.8)	
	Vaginal	833 (74.2)	

*Mean ± Standard Deviation (SD) unless otherwise noted

^†^Includes abnormal placentation, fetal anomaly, intrauterine growth restriction, abnormal non-stress test, abnormal Doppler/ Biophysical Profile findings, reduced fetal movement at term, oligohydramnios, other evidence of fetus in distress that requires early delivery

^‡^Includes advanced maternal age, deterioration of maternal medical condition, gestational or pre-gestational diabetes mellitus, obstetric cholestasis, preeclampsia, pregnancy induced hypertension, post-dates, premature rupture of membranes (preterm and term), raised body mass index, therapeutic low molecular weight heparin

### Missing data

Of the categorical variables, ‘cervical consistency’, ‘position’, ‘effacement’, and ‘fetal station’ had > 10% missing data, and were therefore not included in the development of the model. There were no missing data for other categorical variables (‘gravidity’, ‘parity’ and ‘fetal indication for induction’). For continuous variables, ‘height’, ‘pre-pregnancy weight’, and ‘weight (at-delivery)’, < 10% of data were missing, therefore estimates were generated using imputation modeling. Data on the continuous variable ‘cervical dilation’ was missing for 8.4% of deliveries. This missingness was related to the method of induction, ranging from 13.2% for prostaglandin gels/tablets to 2.1% for artificial rupture of membranes. In addition, an analysis of variance (ANOVA) was performed, and mean cervical dilation was found to be significantly different among the five methods of induction (*p* < 0.001). Therefore, random imputation for missing dilation values was performed conditional on the method of induction. There were no missing data for ‘maternal age’, ‘parity’, ‘gravidity’, ‘fetal indication for induction’, ‘primary method of induction’, ‘gestational age’, and the outcome variable, ‘delivery method’.

### Model development

**Fig. 2:**
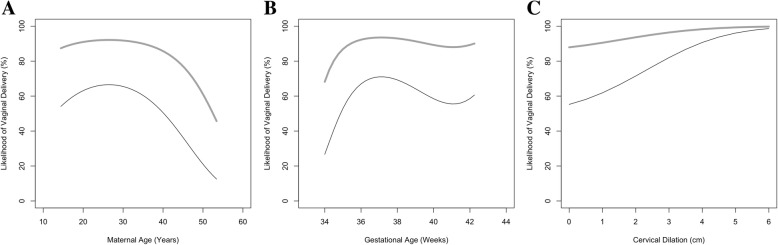
Relationship between maternal age (**a**), gestational age (**b**), cervical dilation (**c**), and likelihood of vaginal delivery, stratified by parity - nulliparous (black-line) vs. multiparous (grey-line)

The 12 predictor variables with ≤10% missing data were entered into a multivariable logistic regression model. The final model included maternal age, gestational age, parity, cervical dilation, pre-pregnancy weight, pre-pregnancy BMI, and weight at delivery (Table 2). The relationship between the likelihood of vaginal delivery with each of maternal age, gestational age, cervical dilation, and parity are illustrated in Fig. 2; for each graph, the value for all other variables are held static as per those found in Table 1. The relationship between the outcome and pre-pregnancy weight, pre-pregnancy BMI, and weight at delivery was not illustrated due to collinearity. For any fixed weights, BMI in the sample takes on only a restricted set of values. Similarly, at the mean pre-pregnancy weights, there is a relatively narrow range of possible delivery weights. It is the trio of values (weight-pre, BMI-pre and weight at delivery), which move together that need to be considered in obtaining predictions.

**Table 2:** Prediction model for probability of successful induction of labour

Variable		β-Coefficient	Standard Error	*p* Value
	(Intercept)	0.82	0.10	< 0.001
a	Parity (Multiparous)	1.79	0.22	< 0.001
b	Weight, pre-pregnancy (kg)	61.21	11.84	< 0.001
	(Weight, pre-pregnancy)^2^	−12.45	7.35	0.09
	(Weight, pre-pregnancy)^3^	−6.48	5.07	0.20
c	BMI, pre-pregnancy (kg/m^2^)	−57.98	8.56	< 0.001
	(BMI, pre-pregnancy)^2^	9.42	5.90	0.11
	(BMI, pre-pregnancy)^3^	12.68	5.04	0.01
d	Gestational Age (weeks)	−6.01	2.66	0.02
	(Gestational Age)^2^	−3.41	2.49	0.17
	(Gestational Age)^3^	4.52	2.47	0.07
e	Weight, at-delivery (kg)	−13.97	7.62	0.07
	(Weight, at-delivery)^2^	7.79	4.90	0.11
f	Dilation (cm)	23.57	3.98	< 0.001
	(Dilation)^2^	4.97	3.87	0.20
g	Maternal Age (years)	−8.43	2.57	< 0.001
	(Maternal Age)^2^	−4.35	2.34	0.06
Equation		Risk Score = 0.82 + [1.79 x a] + [61.21 x b] - [12.45 x b^2^] - [6.48 x b^3^] - [57.98 x c] + [9.42 x c^2^] + [12.68 x c^3^] - [6.01 x d] - [3.41 x d^2^] + [4.52 x d^3^] - [13.97 x e] + [7.79 x e^2^] + [23.57 x f] + [4.97 x f^2^] – [8.43 x g] – [4.35 x g^2^]; probability of vaginal delivery = 1/(1 + e^-Risk Score^)		
Model Performance		Hosmer-Lemeshow goodness-of-fit χ2 = 5.02, 8 degrees of freedom, *p* = 0.76 Area under receiver operating characteristic curve 0.81 [95% CI 0.78–0.83]

### Discrimination, calibration and internal validation

**Fig. 3:**
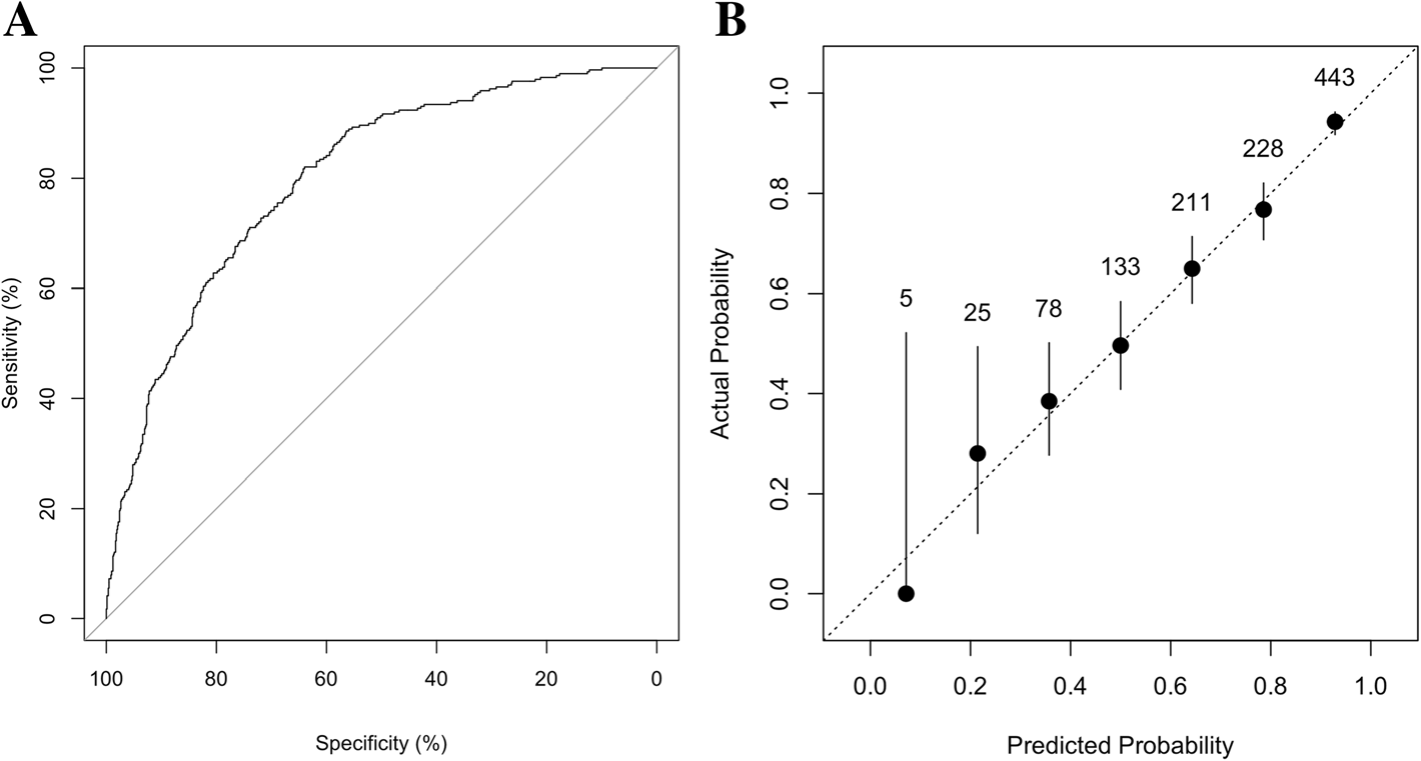
Model Performance [3a – Discrimination; 3b – Calibration] Numbers in Graph B indicate number of labours induced

The prediction model discriminated well between inductions that were successful and those that were not (Fig. 3a, AUROC 0.81 [95% CI 0.78 to 0.83]). The calibration plot for the probability of vaginal delivery showed a good correlation between the predicted and actual probabilities (Fig. 3b, Hosmer-Lemeshow: χ2 = 5.02, *p* = 0.76), except at the lowest predicted probabilities for which numbers were too small to draw meaningful conclusions. Finally, the model performed well in the underlying population as shown by similar performance in the cross-validation procedure (AUROC 0.77 [95% CI 0.73 to 0.82]).

### Sensitivity analysis

The impact of imputation modeling on the developed prediction model was assessed by looking at the performance of the model in the 942 inductions (83.8%) that had complete information for the 12 predictor variables included in the final model. Model performance did not change (AUROC 0.81 [95% CI 0.78 to 0.84]).

We attempted to develop a second model using the pregnancies that had complete information for all 16 predictor variables, as previously described. However, only 257 pregnancies (22.9%) with 49 cesarean deliveries fulfilled this criterion. Therefore, we did not evaluate the performance of the model due to concerns regarding overfitting.

## Discussion

Over the past decade, the indications for inducing labour have continued to increase. Given the clinical-, resource- and cost implications of failed IOL, a number of prediction models aimed at determining the likelihood of vaginal birth following IOL have been developed [10]. However, in order to have widespread applicability, the ideal prediction model must include variables accessible on antenatal history and physical/cervical examination alone, and not on complex radiologic and biochemical parameters [22]. We have developed and validated a clinical prediction model in a large cohort of consecutive labour inductions regardless of parity, cervical favorability, and indication for IOL, with good performance.

This model highlights the importance of demographic variables such as maternal age, maternal weight, parity and gestational age in determining the success of IOL. In the final model, maternal weight was represented by three variables - pre-pregnancy weight, pre-pregnancy BMI and weight at delivery. Although similar, and to an extent collinear, these parameters take into account slightly different characteristics such as maternal height and gestational weight gain, both of which were not in the final model. Also, although collinearity makes estimates of individual coefficients unstable, reliable predictions can still be obtained [19]. In contrast, our model eliminated a number of variables that have been included in earlier models. We were not able to include maternal race as race-based data is not collected in Ontario, and ethnicity in our retrospective dataset was self-reported. We also did not include fetal weight in our model. Although increased fetal weight (macrosomia) is often thought to be associated with failure of IOL, this has not always been shown to be true [23]. In addition, the estimated fetal weight in the third trimester is not always accurate or consistent [24, 25], and as an assessment of fetal weight is not always performed prior to IOL, its inclusion would compromise the usability of the model. We performed post-hoc analysis to look at the distribution of birth weights between those that had cesareans vs. vaginal deliveries, and as anticipated, found no difference in the mean birth weights between the groups (Additional file 3), suggesting that excluding fetal weight from the initial model was not inappropriate. Pelvic adequacy which has been suggested as a risk factor [26], was similarly not included as its assessment is often subjective and not always performed prior to IOL. Maternal height, a surrogate determinate of pelvic adequacy was however included. Although not included in the final model, novel to our study was the assessment of fetal indication for labour induction as a variable, as this is often thought to be associated with failed IOL. The similar rate of fetal indication in both cesarean and vaginal delivery groups (22.4% vs. 24.9%) showed that this variable does not necessarily affect the success of vaginal birth following IOL.

This study has a number of other strengths in addition to the above. It uses rigorous methodology and meets sample size requirements, while building on limitations of previous published models identified in our systematic review [10]. The model was derived used a large dataset that comprised women with high- and low-risk pregnancies, from diverse antenatal and demographic backgrounds, regardless of parity, indication for labour induction and cervical status. The broad inclusion criteria ensures that one model can be used in all women undergoing IOL as opposed to many that are very restrictive in their inclusion criteria and fail to perform adequately in other populations. We have deliberately avoided externally validating this model in a single dataset. A recent model that performed well in one externally validated cohort [23] was shown to perform poorly in another [11]. We believe that external validation therefore should be performed simultaneously in a large number of diverse cohorts, and factors responsible for variation in performance be explored. Finally, our model was robust to a sensitivity analysis and appears not to be overfitted, having similar discriminative ability in the full sample and in the cross-validation exercise.

The main limitation of our study was the proportion of missing data, secondary to its retrospective study design. Although robust to a sensitivity analysis that fitted a model to complete case data, we were unable to investigate the potential predictive value of four variables: cervical effacement, consistency, position, and fetal station, with more than 10% of their values missing. The reason for the high percentage of missing data in these categories in clinical documents is the implied nature of conventional descriptions (e.g. long and closed) for unfavorable cervices, meant to represent an uneffaced, (often) firm, posterior cervix with a high fetal station. It is possible that although variables such as cervical consistency and position may be less likely to influence the prediction of the success of vaginal birth, cervical effacement and fetal station might still be important predictors [27–31]**.** We acknowledge that this is a major limitation that could compromise the model, and will consider these variables during the prospective global validation of this model. Another limitation was the exclusion of women with a previous cesarean delivery due to the low rates of IOL in this population at our center. As women with a prior cesarean form a distinct group in whom the success of IOL depends on factors specific to the prior delivery, this population is probably best excluded while using this prediction model, in favor of a model that takes into account the time since the prior cesarean delivery and emerging evidence on integrity of the previous scar. Another limitation due to the retrospective nature of this study and multiple hand-overs between a large group of obstetricians during the course of labour, is that we were not able to determine whether criteria for failed arrest and induction disorders were met prior to performing the cesarean delivery for these indications [32] or the effect of the care provider on cesarean delivery rates [33]. The subjective nature and lack of universality of these parameters justified their exclusion from a final prediction model that we anticipated would be used in routine clinical practice.

**Fig. 4:**
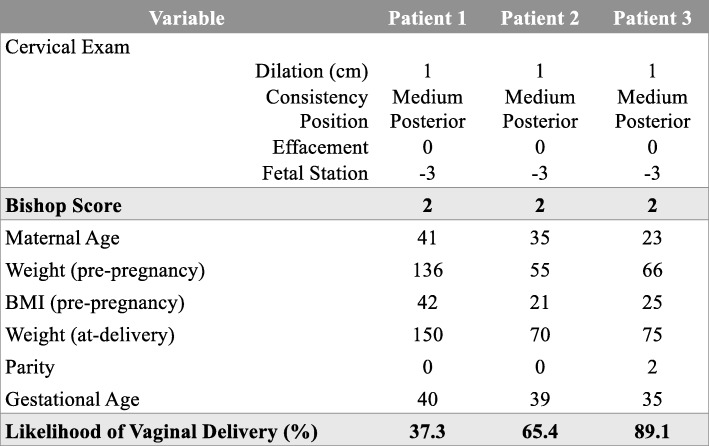
Clinical Applicability

Although the Bishop score was intended to be used as a discriminatory tool, is commonly used to predict the success of IOL [1, 8]. This implies that IOLs are often deferred when the Bishop score is low and possibly offered when the score is high. A systematic review of 157 randomized trials has shown that cervical favorability is not a determinant of the success of vaginal birth following IOL [34]. In fact, there is a suggestion that deferring indicated IOLs in women with unfavorable cervices might result in higher cesarean delivery rates, as their cervices are likely to remain unfavorable when the clinical condition deteriorates, not allowing sufficient time for cervical ripening and a vaginal birth [35]. Our prediction model that takes into account numerous predictor variables in addition to the cervical status, provides clinicians with an alternative to the Bishop score, an important development given the poor predictive ability of the Bishop score and the large number of IOLs performed in contemporary obstetrics. An example of the clinical applicability of our model is demonstrated in Fig. 4. All three patients have an identical Bishop Score of 2, but the model is able to discriminate between patients based on other characteristics, giving patient 1 a 37% chance of success and patient 3 a 89% chance of a successful vaginal birth following IOL. In clinical practice, this has the potential to influence healthcare utilization in a positive manner. For example, a low chance of success, even in the presence of a good Bishop score could alter a decision to defer IOL by a few days in case of a less urgent indication or consider outpatient cervical priming if applicable to improve the chance of success. It could also influence the mode of delivery in cases of severe early-term IUGR, wherein one might feel encouraged about attempting IOL should the chance of success be deemed high enough. Conversely, one might opt for an elective cesarean delivery in a woman with a BMI > 40 based on a poor prediction score to avoid an emergency intrapartum cesarean delivery, which is associated with higher maternal and neonatal adverse events.

It must be added that the potential of prediction to influence decision-making also implies potential for harm, through the dissemination of misinformation at the point of care especially from insufficiently validated models [36]. There is also the potential of misuse of prediction models by women and healthcare providers that deem a certain percentage chance of vaginal birth following IOL as low, and opting for an elective cesarean delivery instead. This may be unavoidable and measures to ensure that women are counselled about the advantages of a vaginal birth and encouraged to attempt IOL should form part of IOL protocols and institutional guidelines. Arguments against the use of prediction models notwithstanding, in this era of personalized medicine, consumers desire information on the chance of success of an intervention, in order to facilitate a fuller discussion on what to expect in the event of failure. The intent of deriving this prediction model, is essentially to enhance this discussion and to enable appropriate allocation of healthcare resources, while reducing cesarean delivery rates through the optimal timing of IOL, which has been shown in numerous randomized trials to lower cesarean rates when compared with expectant management in any population [5, 34]. If used as intended, a well-validated prediction model is unlikely to result in a rise in cesarean delivery rates. However, introduction of any model into clinical practice without assessing the willingness of women and healthcare providers to use it in shared decision making, or without assessing its performance globally and determining factors responsible for variation in its performance could do more harm than good. We therefore would like to exercise caution with regard to the immediate implementation of this, or any other model, unless these planned preliminary studies are conducted.

## Conclusions

We have successfully derived and internally validated a well-performing clinical prediction model for IOL in a large and diverse population using variables readily accessible through maternal demographic data, antenatal history, and cervical examination. Once prospectively validated in varied settings, and if shown to be acceptable to pregnant women and healthcare providers as well as clinically and cost-effective, this model has potential for widespread use in clinical practice and research for enhancing patient autonomy, improving induction outcomes, and optimizing allocation of resources.

## Additional files

Additional file 1: TRIPOD Checklist. (DOCX 90 kb)

Additional file 2: Definitions of variables and outcomes. (DOCX 16 kb)

Additional file 3: Distribution of fetal weight based on mode of delivery. (PNG 130 kb)

## Abbreviations

AUROC (curve): Area under the receiver operating characteristic (curve)
BMI: Body mass index
IOL: induction of labour
