# Supplementary material for: Derivation and validation of a model predicting the likelihood of vaginal birth following labour induction
Source: BMC Pregnancy Childbirth. 2019 Apr 16;19:130. doi: 10.1186/s12884-019-2232-8 (PMC6469110; doi:10.1186/s12884-019-2232-8)
Supplement: Supplementary file 2 — Definitions of variables and outcomes. (DOCX 16 kb) [file 12884_2019_2232_MOESM2_ESM.docx]

Additional file 2: Variable/Outcome Definitions

| **Variable** | **Definition** |
| --- | --- |
| Maternal Age | Age of the mother at time of induction |
| Maternal Height | As reported in antenatal records, in centimeters |
| Maternal Weight (pre-pregnancy) | As recorded at first antenatal visit, in kilograms |
| Maternal Weight (at-delivery) | As recorded in antenatal records, in kilograms |
| Gestational Weight Gain | The difference between pre-pregnancy weight and last recorded maternal weight during pregnancy; in kilograms |
| Body Mass Index (BMI) | [(weight in kilograms)/(height in meters)^2^] |
| Parity | As reported in antenatal records |
| Gravidity | As reported in antenatal records |
| Cervical Exam  Dilatation  Consistency  Position  Effacement  Fetal Station | As reported in delivery records, at the time of induction |
| Primary method of induction | The first method of labour induction as reported in delivery records |
| Fetal Indication for Induction | Any or more than one of: abnormal placentation, fetal anomaly, fetal growth restriction, abnormal non-stress test, abnormal Doppler studies or biophysical profile, reduced fetal movements at term, oligohydramnios, other evidence of fetus in distress that require early delivery |
| Gestational Age | Weeks + days/7; in weeks |
| Mode of Delivery | Cesarean delivery or vaginal delivery |
