# Supplementary figures and images for: Derivation and validation of a model predicting the likelihood of vaginal birth following labour induction
Source: BMC Pregnancy Childbirth. 2019 Apr 16;19:130. doi: 10.1186/s12884-019-2232-8 (PMC6469110; doi:10.1186/s12884-019-2232-8)

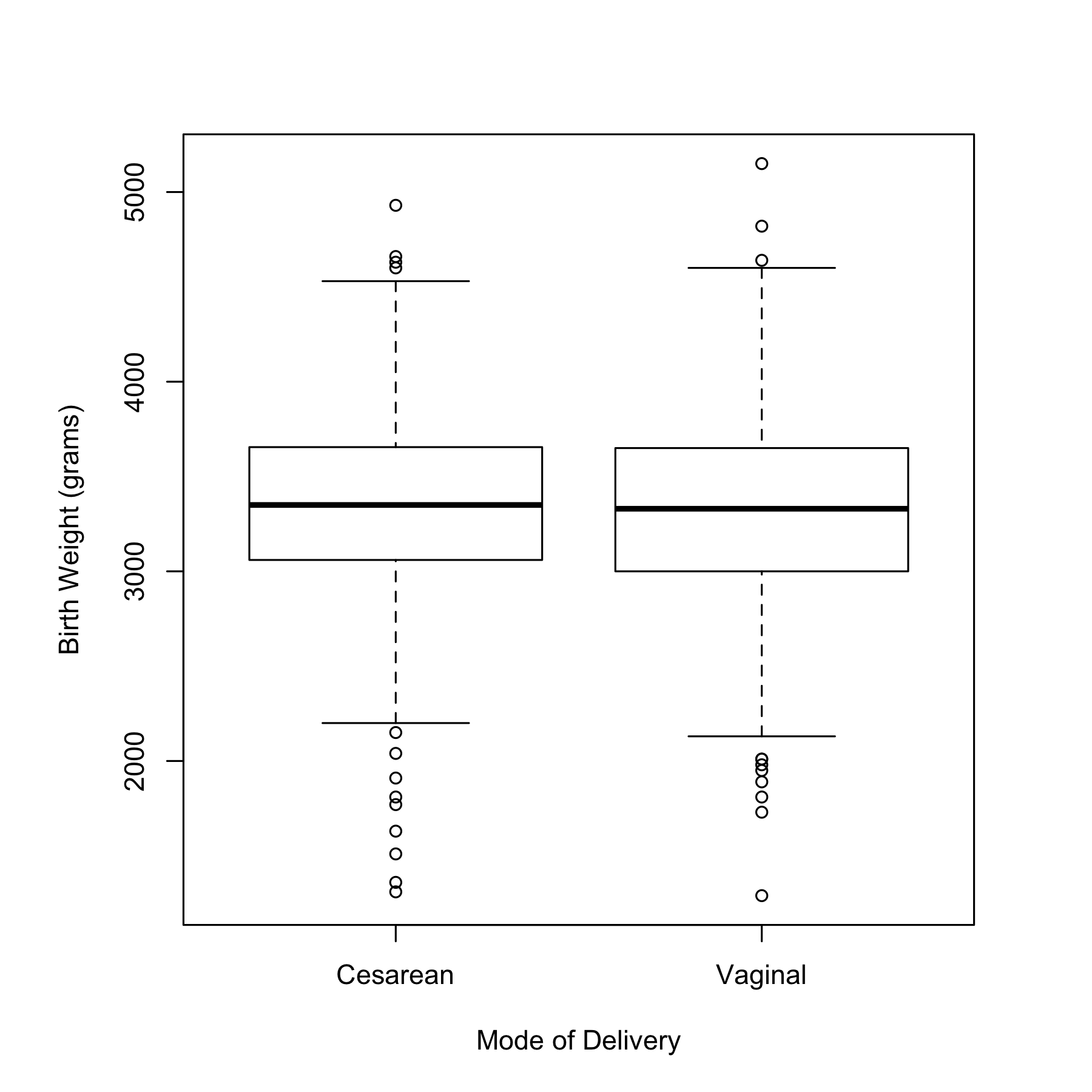

Supplement: Supplementary file 3 — Distribution of fetal weight based on mode of delivery. (PNG 130 kb) [file 12884_2019_2232_MOESM3_ESM.png]
